# CGH analysis in Colombian patients: findings of 1374 arrays in a seven-year study

**DOI:** 10.1186/s13039-018-0398-9

**Published:** 2018-08-22

**Authors:** Mary García-Acero, Fernando Suárez-Obando, Alberto Gómez-Gutiérrez

**Affiliations:** 1 Instituto de Genética Humana, Facultad de Medicina, Pontificia Universidad Javeriana, Bogotá, Colombia; 2 Servicio de Genética, Hospital Universitario San Ignacio, Pontificia Universidad Javeriana, Bogotá, Colombia

**Keywords:** Array CGH, Copy number variation, Unusual finding, Microdeletion syndrome, Microduplication syndrome

## Abstract

**Background:**

Array-based comparative genome hybridization (array CGH) is a first-line test used in the genetic evaluation of individuals with multiple anomalies, developmental delays, and cognitive deficits. In this study, we analyzed clinical indications and findings of array CGH tests of Colombian individuals forwarded to a reference laboratory over a period of seven years in order to evaluate the diagnostic performance of the test in our population.

**Results:**

The results of 1374 array CGH analyses of Colombian individuals were referred to the Andean Reference Institute in Colombia (Instituto de Referencia Andino) during a 7-year period (2009–2015). Chromosomal imbalances were detected in 488 cases (35%), whereas 121 cases were classified as nonpathogenic variants, 65 cases (4.7%) were classified as variants of uncertain significance, and 302 cases (22%) were classified as abnormal or pathogenic. The most common findings in the abnormal and/or pathogenic set were deletions, followed by duplications and complex rearrangements. Variants in the carrier status of autosomal recessive diseases were identified as incidental findings in 29 subjects (2%).

**Conclusions:**

Clinical indications preceding the referral of aCGH in Colombian patients are not standardized and result in unexpected pathogenic variants as well as secondary findings that need careful interpretation. Development of local infrastructure will probably improve the communication between all stakeholders, to ensure accurate clinical diagnoses.

## Background

Genetic diseases are mainly caused by mutations in specific genes (Mendelian conditions) or by chromosomal rearrangements. The latter were initially studied using cytogenetic techniques such as karyotyping. With the advent of array-based comparative genome hybridization (aCGH), the increased detection rate of chromosomal imbalances in the human genome has allowed the diagnosis of syndromic phenotypes with previously unknown etiologies, contributing to our understanding of several neurodevelopmental disorders and to the etiology of congenital abnormalities [1, 2].

aCGH is a comparative test in which two samples are differentially labeled with fluorescent dyes. The hybridization of the labeled genomes to an array, comprising probes spaced along the genome, allows the detection of differences in copy number between the two samples when analyzed using quantitative imaging methods and analytical software to assist in identifying each targeted-DNA sequence [3]. Current aCGH tests detect gain or loss of genomic material > 1 kb in size. Copy number variations (CNVs), a type of genetic variation [4], are considered among the most common causes of human disease [5]. Different criteria can be used to guide the interpretation and clinical relevance of CNVs, including inheritance, size, type, and gene content [6]. Pathogenic CNVs are more frequently found as de novo events, particularly those related to severe disorders that involve neurodevelopment abnormalities associated with congenital malformation. By contrast, some inherited CNVs can cause a severely abnormal phenotype. These can be pathogenic, even when the subject has phenotypically normal parents, and constitute a phenomenon that can be regarded as a CNV with incomplete penetrance [7].

Although there have been increases in both the accuracy and speed of execution of molecular genetic testing, in line with a decrease in test costs and higher availability in the clinical setting, the volume and complexity of the data generated by new molecular techniques have created considerable uncertainty regarding the clinical value of the results obtained [8]. Since the diagnostic performance of aCGH has been established to be between 15 and 20% in patients with neurodevelopmental delays and congenital anomalies [9–11], it is necessary to continuously analyze the results of the test by considering CNVs in large cohorts of patients. In this study, we describe of results of 1374 aCGH analyses that were handled in a reference laboratory in Colombia, South America, during the period 2009–2015, characterizing the indications of reference, pathogenic variants, and secondary findings.

## Methods

Instituto de Referencia Andino (IRA) in Bogotá, D.C., Colombia, is a national reference laboratory that receives referred samples from across the country, and also some samples from Ecuador and Panamá. During the period from 2009 to 2015, the IRA received 1374 different blood samples to report on the corresponding aCGH analyses. All the blood samples were immediately referred to the Medical Genetics Laboratories at Baylor College of Medicine (MGL-BCM) in Houston, Texas, USA, as part of a formal agreement. The primary molecular analysis was accordingly performed at MGL-BCM using aCGH with approximately 180,000 oligos covering the whole genome at an average resolution of 30 kb, and including 1714 genes with all exons covered, 700 microRNAs, and the entire mitochondrial genome. For the array, DNA labeling and hybridization were performed according to the manufacturer’s protocol (Agilent Technologies). arrays were scanned on an Agilent G2565 scanner and image files were quantified using Agilent’s Feature extraction software (V9.0). The IRA database of aCGH test results includes variables such as the age and sex of the patient and, in some cases, the medical indication for test referral. Here, we list the frequencies and specificities of the findings of 1374 aCGH tests conducted over a 7-year period. Furthermore, with the purpose of determining the clinical relevance of the corresponding results, we analyzed each of the reported analyses, comparing, where possible, the initial clinical impression for referral with the reported result. We looked for evidence relating to the aCGH results and information to evaluate of pathogenicity of CNV in public databases, such as OMIM, ClinVar https://www.ncbi.nlm.nih.gov/clinvar/, dbVar https://www.ncbi.nlm.nih.gov/dbvar/, DECIPHER https://decipher.sanger.ac.uk/, Unique https://www.rarechromo.org/, Chromosomal Variation in Man https://www.ncbi.nlm.nih.gov/books/NBK105441/ and also in the published literature (PubMed), particularly in the cases of uncertain results or incidental findings. Except where specified, results are presented as relative frequencies based on the total number of cases.

## Results

We analyzed data obtained from 1374 array analyses performed for Colombian individuals who were referred to the IRA during a 7-year period. The median age for the group was four years old (range: 0–57 years). The motive for referral was mainly to test for neurological disorders (45%), including a history of developmental delay, epilepsy, autism, cognitive deficits, cerebral palsy, and confirmation percentages of of several genetic syndromes. A secondary reason for referral for the aCGH test was to test for multiple congenital anomalies (26%). A further 5% of referrals were sent for testing clinically defined syndromes, where most of these were referred based on an abnormal karyotype. In 329 cases (24%), there was no specific written indication of the reason for test referral.

In most cases, physician’s written references for the test were notably unspecific. Neuromuscular pathologies include a broad clinical spectrum of conditions such as epilepsy, developmental delay, cognitive impairment, and additional suspicions of Down, Rubinstein-Taiby, Wiliams, Di George, Klinefelter, and Cri du Chat syndromes. Of the 577 cases that were referred with a diagnosis of a neuromuscular abnormality (i.e., 42% of the total samples), the diagnostic yield, which is defined as the ability of the detection test to identify the condition in affected subjects, was just 22%. The second reason for test referral was associated with congenital anomalies (290 cases), in which the diagnostic yield was 28%, corresponding to 81 individuals with CNV of pathogenic significance that explained the phenotype. Twelve patients with a congenital heart defect were referred for aCGH and, among these, the test provided results for diagnosis in only two instances, one with a finding of copy number loss of chromosome band 16p13.3, including multiple exons of the *TSC2* gene associated with Tuberous sclerosis-2, and the other with copy number loss within chromosome band Xp21.1, which encompasses part of the *DMD* gene.

In 54 cases, physicians indicated the test in instances where a previous karyotype was abnormal, in order to either confirm or shed more light on the previous. Subsequent aCGH analyses confirmed the chromosomal abnormality in 34 of these cases (63%).

**Fig. 1:**
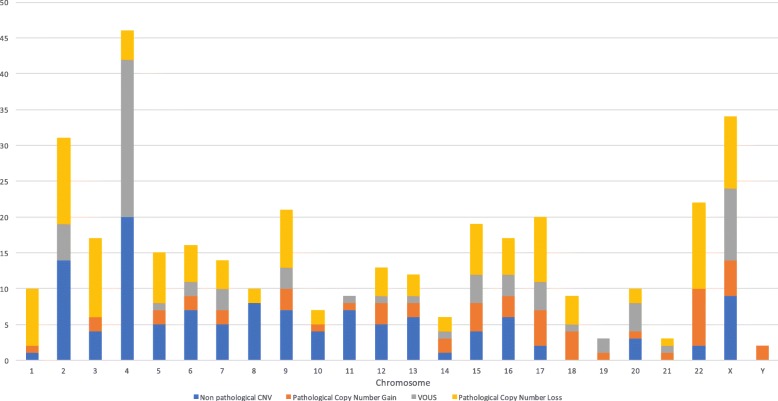
The number of CNV and its pathological implications, as distributed on each chromosome

Imbalances were detected in 488 cases (35%), 121 of which were classified as nonpathogenic variants, including 29 cases (2%) of heterozygous variants of carrier status, 302 cases (22%) classified as abnormal or pathogenic, and 65 cases (4.7%) classified as VOUS [variant of uncertain (or unknown) significance]. The number of CNV found in each chromosome and their pathological implications, are shown in Fig. 1. Among these imbalanced cases, 42 variants were inherited from one of the parents (3%). The most common findings in the abnormal and/or pathogenic sets of cases were 180 deletions (13%), 74 cases of duplication (5.3%), 48 complex rearrangements (3%) defined as structural rearrangements with exchange of genetic material between two or more chromosomes, and four cases with both deletion and duplication (0.3%) (Table 1). Information about nature/classification of deletions and rearrangements regarding size and content are the matter of a subsequent analysis in our Institute of Human Genetics in Bogotá.

**Table 1:** Summary of findings from array CGH testing, Colombia, 2009–2015

Reports	n	Diagnostic yield (%)
	1374	
Abnormal	488	35.9%
Normal	886	64%
Inheritance analysis	76	15% of all abnormal aCGH
De novo	34	45% of variants assessed for heredity
Inherited	42	55% of variants assessed for heredity
Molecular anomalies	488	Percentage of total abnormal imbalances
Deletions		
All chromosomes	246	50.4% of total imbalances
Autosomes	217	44.4% of total imbalances
Sex chromosomes	29	5.9% of total imbalances
Duplications		
All chromosomes	190	39% of total imbalances
Autosomes	164	33.6% of total imbalances
Sex chromosomes	26	5.3% of total imbalances)
Multiple chromosome rearrangements	52	10.0% of total imbalances
Pathogenic imbalances	302	61.8% of all imbalances
Deletions (all chromosomes)	180	59.6% of pathogenic imbalances
Duplications (all chromosomes)	74	24.5% of pathogenic imbalances
Rearrangements	48	15.9% of pathogenic imbalances
Susceptibility loci (carriers)	29	5.94% of total imbalances

Variants of the carrier status of autosomal recessive diseases were identified as incidental findings in 29 subjects (2%), including nine cases of juvenile nephronophthisis, three cases of Bartter syndrome, two cases of dihydropyrimidine dehydrogenase deficiency, and two cases of hyper-IgE syndrome (DOCK8) (Table 2). Other cases were those of unique carriers for a disease. One case was an incidental finding of CNV of the *APC* gene associated to Lynch Syndrome, condition not associated with the indication of the test. The inheritance status of a CNV was determined in 76 subjects (5.5%) and their parents. In only 3% of these cases was one of the parents revealed as a carrier of the patient’s variant.

**Table 2:** Copy number variation of single alleles in genes of autosomal recessive inheritance pathologies: “carrier status”

OMIM	Syndrome	Chromosomal region	n	del (×1)	dup (×3)
256,100	Juvenile nephronophthisis	2q13	9	9	
241,200	Bartter syndrome 2	11q24.3	3	3	
274,270	Dihydropyrimidine dehydrogenase deficiency	1p21.3	2	2	
611,432	hyper-IgE syndrome	9q24.3	2		2
116,920	Leukocyte adhesion deficiency, type I	21q22.3	1	1	
253,600	Muscular dystrophy, limb-girdle, type 2	15q11.1	1	1	
220,290	Deafness, autosomal recessive 1A	13q12.11	1	1	
271,900	Canavan disease	17p13.2	1	1	
615,419	Neuroaxonal neurodegeneration, infantile, with facial dysmorphism	13q33.1	1	1	
610,356	Retinal cone dystrophy 3B	9p24.2	1	1	
231,300	Primary congenital glaucoma	2p22.2	1	1	
613,254	Tuberous sclerosis-2	16p13.3	1	1	
613,826	Leber congenital amaurosis 6	14q11.2	1	1	
210,900	Bloom syndrome	15q26,	1	1	
614,072	Hermansky-Pudlak syndrome-3	3q24	1	1	
609,254	Senior-Loken syndrome	3q13.3	1	1	
201,400	ACTH deficiency	1q24.2	1		1

Regarding non-pathogenic or VOUS cases, we compared the reported status based on laboratory tests with recent reports published in the literature, and in eight cases we found that the status of the variant had changed from a VOUS to a variant with clinical significance, as described in recent reports (Table 3).

**Table 3:** Variants which changed from non-pathogenic or VOUS to a variant with clinical significance on reanalysis

MGL-BCM reported	n	Year of release	Evidence	Reference
GAIN of chromosome band 3p26.3 spanning approximately 0.808 Mb in a non-disease-associated region	1	2015	Duplication of the *CNTN6* gene is associated with a wide spectrum of neurodevelopmental behavioral disorders	• Hu et al. (2015). Journal of Neurodevelopmental Disorders 7:26 • Chunyang (2016). Mol Cytogenet 9; 51 • Mercati et al. (2017). Molecular Psychiatry 22:625–33
GAIN of chromosome band 8q11.23 spanning approximately 0.649 Mb in a non-disease-associated region	2	2015	Duplications in *RB1CC1* as a risk factor for schizophrenia	• Degenhardt (2013). Translational Psychiatry 3(11):e326
LOSS of chromosome band 8p11.21 spanning approximately 0.002 Mb in a non-disease-associated region.	1	2014	*CHRNB3* mediates fast signal transmission at synapses, and may therefore be associated with psychomotor developmental delay	• Miya (2012). Gene 506(1):146–9
LOSS of chromosome band 3p21.1 spanning approximately 0.0052 Mb in a non-disease-associated region.	1	2011	The existence of a tumor-suppressor gene that plays a critical role in the development and progression in various solid malignancies	• Li (2013). PLoS ONE 8(4):e60027 • Lovrecic (2016). Mol Syndromol. 7(2): 93–98
LOSS within chromosome band 20p12.1 spanning approximately 0.007 Mb in a non-disease-associated region.	1	2015	It has been reported that this macrodomain (MACROD2) is expressed in the ventricular zone of the brain, and is associated with several neurologic and psychiatric disorders	• Frye (2016). N A J Med Sci. 9(1):35–37
LOSS of chromosome bands 2q24.2q24.3 spanning approximately 1.054 Mb in a non-disese-associated region.	2	2011	KCNH7 contributes to benign familial neonatal seizures	• Okumura (2011) Epilepsia 52(7):e66–e69 • Belengeanu V (2014) Gene 539(1):168–172, 2014.

## Discussion

CNVs detected by chromosomal microarray analysis or aCGH make a significant contribution to establishing the etiology of neurodevelopmental disorders and congenital anomalies [1, 2, 12]. Although the indication for referring a patient for an aCGH test has previously been defined in the literature [13], in the light of recent methodological advances and redefinition of the pathognomonic significance of the chromosomal anomalies eventually detected, it is necessary to periodically review the diagnostic yield of this molecular technique [11]. Such periodic review is particularly important in developing countries where the infrastructure of genetic laboratories is, in many cases, still relatively rudimentary [14], and also where healthcare insurance imposes access barriers for patients [15].

In this study, nearly 40% of individuals who were referred for aCGH analysis had a chromosomal variant, and 22% of these variants were classified as pathogenic. A similar or even lower diagnostic yield has been achieved in other studies. For example, Ahn et al. [11] found that when 8794 subjects were assessed for developmental delay, dysmorphism, neurodisability, and congenital abnormalities, 25% had abnormal findings in aCGH tests. Similarly, Barnik et al. showed a diagnostic yield of 33% for a group of children with intellectual disability [16], whereas Ho et al. reported a detection rate of 29.4% in their neurodevelopmental cohort of 5487 patients [2]. Further, Kaminsky et al. identified 17.1% pathogenic CNVs in a group of 15,749 individuals who were referred for diagnostic array testing with abnormal clinical phenotypes, including developmental delay/intellectual disability, autism spectrum disorders, and/or multiple congenital anomalies [5].

Deletions account for up to 60% of pathogenic imbalances. Ahn et al. identified deletions in 46% of cases [11], whereas Battaglia et al. reported that the ratio of the occurrence of deletions to duplications was 1.7:1 [17]. Consistently, in our study, the deletion:duplication ratio was 1.26:1. The analysis of deletion/duplication is highly relevant for genotype–phenotype correlation, since deletions are generally more significant than duplications as stated in a previous general review [2]. As the size of a deletion defines the number of affected genes [18], depending on the resolution of the corresponding platform, the exact sizes of the corresponding CNV can be characterized and define a specific syndrome [19]. One of the reasons why deletions have more effect than duplications, is because some genes require two copies for its normal expression. Therefore, if a copy is deleted and only one allele remains, a mutant phenotype might result in a haploinsufficiency of the gen. The CNV number was not proportional to the size of the chromosomes, for example the acrocentric and submetacentric small chromosomes of the group D and E, respectively, had higher number of CNV than the medium submetacentric chromosomes of the group C despite their smaller size. Also the distribution shows that chromosome 22, despite being small acrocentric has a high number of CNV, which has been associated to microdeletion and microduplication syndromes. The incidence of deletion 22q11 has been estimated at one per 4000 live births, placing this syndrome among the most frequent causes of genetic syndromes and as the most common microdeletion human syndrome [20].

In the present study, we found that the reasons for referral, when stated, appeared to follow the recommendations of the American College of Medical Genetics and Genomics (ACMG) [21] for performing aCGH as a first-line test in the postnatal evaluation of individuals with non-specific multiple anomalies, developmental delay, cognitive deficits, or apparently non-syndromic autism spectrum disorders. However, a significant proportion of patients (21%) referred for the test did not have any description of the corresponding clinical presentation or the specific clinical indication of the array. This fact is particularly relevant since the likelihood of determining a given clinical implication of a variant appears to increase if the patient has phenotypic clues that enable normal and abnormal variants to be distinguished [22]. Registering a complete clinical phenotype for interpretation of a molecular anomaly is essential for collecting new evidence to define clinical significances associated with CNVs that are defined as VOUS [23].

Given the high proportion of VOUS and regarding the importance of genetic counseling, the heredity status of a variant is highly relevant. However, in the present study, we found that in very few cases (13% of abnormal aCGH) the carrier status of the parents was evaluated. Among the studied trios, we found that approximately 3% of the pathological variants were inherited. In contrast, Battaglia et al. found that 45% of the identified CNVs were inherited [17]. The analysis of parental status has also been found to be problematic in other published series, as Ahn et al. reported that only 50% of the assessed patients completed inheritance studies [11]. Targeted studies with smaller cohorts probably have an increased likelihood of defining parental status, particularly if molecular strategies such as multiplex ligation-dependent probe amplification (MLPA) are used to confirm results [24]. In our study, the low confirmation of inheritance status may be related to the fact that in Colombia health insurance only covers the test for the proband.

Given the possibility of scanning the whole genome using aCGH, this technique may reveal incidental and unexpected information related to a specific patient or his/her parents [25, 26]. In the present study, we identified 29 cases of carriers of various genetic diseases, none of which were related to a clinical phenotype. However, aCGH information can imply future reproductive decisions or even the development of symptoms associated with the carrier status. The decision regarding whether to disclose the results must be based on clinical significance, the medical intervention that can be used prevent or treat the disease, and the patients’ participation in the decision [27]. However, the disclosure of information must be a decision process that begins during pre-test counseling [28]. In the present study, we did not have access to the eventual specific information on the counseling process in the described cases. However, given the fact that genetic counseling is not a recognized career in Colombia, and that there are relatively few clinical geneticists, who are mainly based in large cities [14], it is reasonable to assume that the disclosure of information relating to incidental findings is not a planned process. In many of the presented cases, no strict regulations were followed. For example, one of the patients analyzed in the present study, a child with neurodevelopmental delay, in whom a deletion of a region that involves the *APC* gene was found and which is associated with the Lynch syndrome, modifies the clinical approach and follow-up, in him and in one of the parents in whom the deletion was confirmed, for a different reason to the indication of the test.

Finally, the updating the CNV status from a VOUS to a clinically significant variant is also a part of the diagnostic and genetic counseling process. However, it requires that laboratories continually monitor the updating of CNV databases and keep abreast of the recent literature in order to assess the status of each patient related to the clinical significance of his/her molecular anomaly [29]. Moreover, laboratories must maintain continuous communication with the referring physicians to report the updates for an appropriate testing and a successful correct diagnosis.

On the basis of our retrospective analysis, we were able to update the CNV status of eight patients. The clinical relevance of their molecular findings was published after the reports had been released to the concerned physicians. However, we do not have any information as to whether the update process was carried out by the laboratory or if the referring physicians changed a clinical decision based on the updated status of the detected CNV. In this regard, the communication process among international reference laboratories and physicians is generally hampered by transnational determinants.

## Conclusions

Based in our retrospective analysis in Colombian patients, we have highlighted the importance of clinical and laboratory interaction. In our analysis a significant portion of patients (24%) were included without any clinical indication information. This fact illustrates a problematic diversity of existing recommendations in Colombia and other countries. Therefore, it is important to discuss and standardize genetic, ethical, legal and economic issues at international level, including the development of uniform quality standards in aCGH processing from the indication of the test, pre-test counseling for secondary findings and uniform interpretation in order to ensure the conditions for international comparability of such studies.

Clinical indications preceding the referral of aCGH in Colombian patients in Colombia, are not standardized and result in unexpected pathogenic variants as well as secondary findings that need careful interpretation. Development of local infrastructure will probably improve the communication between all stakeholders. However, local laboratory services must comply with international standards and the quality of local genetic testing, which has been a cause for concern, needs to be addressed [30].

The use of aCGH analysis poses certain challenges. Careful and prudent indication for the test, aimed at determining a specific etiology, must be accompanied by pre- and post-test counseling given the implications of results not being associated with the phenotype, the modification of reproductive decisions, and patient surveillance and clinical attention.

With regards to test implications, aCGH test report should include a careful review of its meaning at the time of analysis and over time, given the continued contribution of literature and functional assays to the significance of variants associated with different phenotypes. Findings in this test can potentially determine etiology, highlight the need to modify the genetic counseling of individuals with carrier status, or indicate unusual results or unknown significance; however, knowledge of clinical indications is necessary to ensure an accurate diagnostic yield.

## Abbreviations

aCGH: Array comparative genomic hybridization
CNV: Copy number variation
IRA: Instituto de Referencia Andino
MGL-BCM: Medical Genetics Laboratories at Baylor College of Medicine
VOUS: Variant of uncertain significance
